# Hyperoxia during extracorporeal cardiopulmonary resuscitation for refractory cardiac arrest is associated with severe circulatory failure and increased mortality

**DOI:** 10.1186/s12872-021-02361-3

**Published:** 2021-11-14

**Authors:** Jean Bonnemain, Marco Rusca, Zied Ltaief, Aurélien Roumy, Piergiorgio Tozzi, Mauro Oddo, Matthias Kirsch, Lucas Liaudet

**Affiliations:** 1 The Service of Adult Intensive Care Medicine, Lausanne University Hospital and University of Lausanne, Rue du Bugnon 46, 1011 Lausanne, Switzerland; 2 The Service of Cardiac Surgery, Lausanne University Hospital and University of Lausanne, Rue du Bugnon 46, 1011 Lausanne, Switzerland

**Keywords:** Extracorporeal cardiopulmonary resuscitation (ECPR), Veno-arterial membrane oxygenation (VA-ECMO), Cardiac arrest, Hyperoxia

## Abstract

**Background:**

High levels of arterial oxygen pressures (PaO_2_) have been associated with increased mortality in extracorporeal cardiopulmonary resuscitation (ECPR), but there is limited information regarding possible mechanisms linking hyperoxia and death in this setting, notably with respect to its hemodynamic consequences. We aimed therefore at evaluating a possible association between PaO_2_, circulatory failure and death during ECPR.

**Methods:**

We retrospectively analyzed 44 consecutive cardiac arrest (CA) patients treated with ECPR to determine the association between the mean PaO_2_ over the first 24 h, arterial blood pressure, vasopressor and intravenous fluid therapies, mortality, and cause of deaths.

**Results:**

Eleven patients (25%) survived to hospital discharge. The main causes of death were refractory circulatory shock (46%) and neurological damage (24%). Compared to survivors, non survivors had significantly higher mean 24 h PaO_2_ (306 ± 121 mmHg vs 164 ± 53 mmHg, p < 0.001), lower mean blood pressure and higher requirements in vasopressors and fluids, but displayed similar pulse pressure during the first 24 h (an index of native cardiac recovery). The mean 24 h PaO_2_ was significantly and positively correlated with the severity of hypotension and the intensity of vasoactive therapies. Patients dying from circulatory failure died after a median of 17 h, compared to a median of 58 h for patients dying from a neurological cause. Patients dying from neurological cause had better preserved blood pressure and lower vasopressor requirements.

**Conclusion:**

In conclusion, hyperoxia is associated with increased mortality during ECPR, possibly by promoting circulatory collapse or delayed neurological damage.

**Supplementary Information:**

The online version contains supplementary material available at 10.1186/s12872-021-02361-3.

## Background

Extracorporeal cardiopulmonary resuscitation (ECPR) for refractory out-of-hospital (OHCA) or in-hospital (IHCA) cardiac arrest has gained growing interest over the past years [1, 2]. However, recent multicenter registries reported relatively low favorable neurological outcome and survival in heterogeneous cohorts of patients treated with ECPR [3, 4], implying that this strategy should be limited to highly selected patients [4–6]. Besides patient selection, improving the quality of post-cardiac arrest care might also be key to improve ECPR outcome. The restoration of systemic oxygenated blood flow after cardiac arrest may trigger reperfusion injury, largely mediated by the generation of oxidants and free radicals [7], whose magnitude may be influenced by the arterial partial pressure of oxygen (PaO_2_) during reperfusion [8, 9]. In conventional CPR, an association between hyperoxia (defined as supranormal PaO_2_ caused by high fractions of inspired oxygen, with a cut-off value of 300 mmHg frequently used in clinical studies) after return of spontaneous circulation (ROSC) and outcome has been indeed reported in large observational studies [10, 11].

Patients undergoing ECPR may be exposed to significant hyperoxia, given the ease to oxygenate blood through the oxygenator [12], and recent retrospective analyses indicated that hyperoxia during the first 24 h of ECPR was associated with reduced survival [13, 14]. One of these studies reported a significant association between circulatory shock during the first 24 h and mortality [14], whereas another study showed that most deaths (88%) occurred during the first 48 h and were related to multiple organ failure [13]. These findings suggest the hypothesis that early hyperoxia during ECPR may precipitate cardiovascular dysfunction leading to organ failure and death, by exacerbating post-resuscitation disease related to reperfusion injury. To address this hypothesis, we sought to determine the potential association between hyperoxia during the first 24 h of ECPR and the severity of hemodynamic failure, as well as its possible association with mortality in a cohort of 44 patients treated with ECPR for refractory IHCA and OHCA. In addition, since hyperoxia-mediated toxicity is largely related to the generation of oxygen free radicals, one can hypothesize that a longer duration of hyperoxia might be associated with a cumulative exposure to such species, hence greater toxicity, as recently shown by Roberts et al. in a prospective study on hyperoxia in cardiac arrest [11]. Therefore, instead of relying on a single value of PaO_2_, we averaged the values of several PaO_2_ measurements during the first 24 h of ECPR, in order to take into account the duration of exposure to elevated PaO_2_ levels during this period of time. We then evaluated a possible association between PaO_2_ levels, circulatory failure and death during ECPR.

## Methods

### Study setting

This study was approved by our ethical committee (Commission Cantonale d’Ethique de la Recherche sur l’Etre Humain/CER-VD-Nr: 2017–01,184), as a retrospective use of clinical data with waiver of consent (CER-VD-Nr: 2017–011,184), and conforms to the STROBE statement for the report of observational studies. All methods were performed in accordance with the relevant guidelines and regulations. The cohort included 44 consecutive patients hospitalized in our 35-bed multidisciplinary ICU for refractory, non-traumatic and non-hypothermic, OHCA and IHCA treated with ECPR from June 2017 to June 2019. During this period, our internal recommendations for starting ECPR were: refractory OHCA or IHCA; no flow < 5 min; low flow < 80 min; age < 75 years; absence of major co-morbidities; absence of Do Not Resuscitate order. A subset of patients of the current cohort were also included in an unrelated study evaluating automated pupillometry during Veno-Arterial ECMO (VA-ECMO) for various indications [15].

### Treatment strategy

The insertion of venous and arterial femoral cannulas was performed in the hospital by a cardiac surgeon. Initial VA-ECMO settings included a sweep fraction of oxygen (F_S_O2) of 100% and a pump setting for a target blood flow of 40–60 ml/kg, using the Maquet Cardiohelp ECLS system®. Sweep gas flow was adapted to maintain normal PaCO_2_. Systemic anticoagulation (intravenous heparin) initiated at the end of surgery was maintained to achieve an anti-Xa activity of 0.3–0.5. A coronary angiography was performed at the discretion of the in charge physicians, when an ischemic origin of cardiac arrest was strongly suspected, according to internal recommendations. Noradrenaline (NA) and Adrenaline (Adre) were given to maintain mean blood pressure (BP) ≥ 65 mmHg. Intravenous (IV) fluids were administered to maintain the target blood flow. Mechanical ventilation was performed with a tidal volume of 6 ml/kg, rate of 10–15/min and PEEP of 6–10 cm H_2_O. Both FiO_2_ and FsO_2_ were set by default at 100% on ECPR initiation. Following these initial settings, decisions to reduce FiO_2_ or/and FsO_2_ were entirely at the discretion of the treating physicians, without specific recommendation. Sedation was maintained with Propofol (2–4 mg/kg/h) or Midazolam (0.05–0.15 mg/h). Targeted temperature management at 35–36 °C for 24 h was implemented in all patients. Criteria for discontinuing VA-ECMO were the absence of cardiac function recovery, intractable circulatory shock, or evidence of severe neurological injury (major stroke on cerebral imaging or evidence of severe anoxic brain injury according to a multimodal outcome prediction, combining neurological examination, electrophysiological data, and plasma levels of serum neuron-specific enolase (NSE), as described in [16]). Criteria for weaning of VA-ECMO included a mean BP > 65 mmHg, left ventricle ejection fraction > 20% and aortic velocity time integral (VTI) > 10 cm on transthoracic echocardiography, under minimal vasopressor (Norepinephrine ≤ 0.1 μg/kg/min) and inotropic (Dobutamine < 4 μg/kg/min) support and ECMO flow (1L/min).

### Data collection

Demographic variables included age, sex, location of CA (OHCA, IHCA), initial rhythm, duration of no flow and low flow (total duration of CPR before ECMO initiation), SAPS 2 score, ICU and hospital length of stay, duration of ECMO treatment, proportion of patients undergoing coronary angiography and angioplasty (PTCA), as well as the causes of death.

Hemodynamic variables included: (A) Mean arterial blood pressure (mean BP, obtained via an arterial catheter in all but 3 patients), from which we computed the average from all measurements performed each 2 h during the first 24 h (or until death if it occurred before 24 h). (b) Pulse pressure (systolic minus diastolic blood pressure), determined during the first 24 h as an indirect indicator of native cardiac output recovery [17], which was averaged from values obtained at 2, 6, 12 and 24 h (or until death if it occurred before 24 h). (C) The amount of catecholamines, fluids, packed red blood cells and fresh frozen plasma administered (first 72 h).

Blood gas data were obtained from an indwelling intra-arterial catheter, whose position was recorded (right radial, left radial or femoral). The values of PaO_2_ measured at 5 different times of ECPR (first 15 min, 2 h, 6 h, 12 h and 24 h) were averaged as the mean 24 h PaO_2_ (in patients dying before 24 h, mean PaO_**2**_ was computed from values obtained during the time spent under ECPR until death). Arterial blood lactate was determined in the first arterial blood sampling (first 15 min of ECPR).

### Data analysis

Continuous variables are expressed as means ± SD, or medians and interquartile ranges (IQR), and categorical data as absolute numbers and percentages. All comparisons were done using the Wilcoxon’s rank sum test for continuous variables and the chi-square test for categorical variables. We determined which variables were associated with survival using univariate logistic regression analysis for continuous variables and contingency analysis with Pearson’s test for categorical variables. To evaluate the association of hyperoxia with mortality, multivariable logistic regression was applied. We first considered a model including only co-variables significantly associated with mortality in univariate analysis (low flow duration, first arterial lactate, mean BP and mean PaO_2_). We next applied a model including co-variables commonly considered as important clinical predictors of poor outcome, including age, low flow and no flow duration, shockable rhythm, the duration of ECMO, as well as mean PaO_2_. In both models, hospital mortality was the response binomial variable. Odds ratios (ORs) with 95% CI were calculated (for continuous variables, ORs were calculated per unit change of each variable), and Wald statistics was used to assess the significance of each variable. Furthermore, due to the relatively low number of events in our cohort and the inherent risk of overestimating regression coefficients [18], we complemented this analysis by running several logistic regressions using only mean PaO_2_ and a second co-variable at a time (including co-variable with a p value < 0.2 in the multivariable logistic regression: no flow, low flow, and shockable rhythm). To control for possible type I error, we introduced a Bonferroni adjustment for assessing significance in these 3 models (thus, a p value of 0.05/3 = 0.016 was used as the significance limit in these analyses). To determine a possible association between PaO_2_ and circulatory failure, we performed bivariate analyses and simple linear regressions between mean PaO_2_ and mean blood pressure and between mean PaO_2_ and the amount of vasopressive catecholamines administered, with calculation of the Pearson r coefficient. To determine the influence of the arterial catheter position on PaO_2_, PaO_**2**_ values obtained in the different catheter positions were compared using Wilcoxon rank test. The JMP software, version 15, was used for all the analyses, and a p value < 0.05 was considered statistically significant.

## Results

The characteristics of patients and differences between survivors and non survivors are shown in Table 1. No patient in our cohort displayed ROSC before ECMO initiation. For the whole cohort, in-hospital survival was 25% (11pts), including OHCA: 7/27 = 26% and IHCA: 4/17 = 24% (p = 0.86). Survivors had lower SAPS 2 scores, longer LOS and ECPR duration. There were no differences in terms of location of CA, initial rhythm, no flow duration and PaCO_2_ levels, whereas low flow duration, first arterial lactate and intravenous fluids administration were significantly higher in non survivors.

**Table 1:** Characteristics of patients

	All pts (n = 44)	Non survivors (n = 33)	Survivors (n = 11)	p value
Age (years), mean (SD)	52 (14)	54 (12)	48 (19)	0.456
Male sex, n (%)	31 (70)	24 (73)	7 (63)	0.567
SAPS 2, mean (SD)	83 (13)	85 (14)	76 (6)	0.003
ICU LOS (days), median (IQR)	2.6 (18.8)	1.5 (4.3)	22.9 (28.8)	< 0.001
HOSP LOS, (days), median (IQR)	2.8 (27.3)	1.6 (4.2)	51.4 (38.0)	< 0.001
ECMO Weaning, n (%)	15 (34)	4 (12)	11 (100)	< 0.001
Survival (Hospital), n (%)	11 (25)	0 (0)	11 (100)	
ECMO duration (hours), median (IQR)	43 (106)	28 (106)	81 (74)	0.024
Location of CA				0.858
OHCA, n (%)	27 (61)	20 (65)	7 (63)	
﻿IHCA, n (%)	17 (39)	13 (35)	4 (37)	
Initial rhythm				0.141
﻿VF, n (%)	23 (52)	16 (49)	7 (64)	
﻿PEA, n (%)	14 (32)	11 (33)	3 (27)	
﻿Asystole, n (%)	6 (14)	6 (18)	0 (0)	
﻿Unknown, n (%)	1 (2)	0 (0)	1 (9)	
﻿Shockable Rhythm	23 (52)	16 (48)	7 (64)	0.214
No flow (minutes), mean (SD)	1.2 (2.0)	1.1 (2.0)	1.5 (2.2)	0.572
Low flow (minutes), mean (SD)	68 (23)	73 (22)	51 (18)	0.005
Mean PaO_2_ (first 24 h, mmHg), mean (SD)	269 (124)	306 (121)	164 (53)	0.001
Mean PaCO_2_ (first 24 h, mmHg), mean (SD)	37 (6)	36 (7)	38 (3)	0.525
First lactate (mmol/L), mean (SD)	13.4 (5.9)	14.9 (5.8)	9.4 (4.1)	0.004
Mean BP (first 24 h), mean (SD)	67 (12)	64 (12)	75 (5)	0.007
Pulse Pressure (first 24 h), mean (SD)	20 (13)	18 (12)	25 (13)	0.149
Coronarography, n (%)	22 (50)	16 (48)	6 (54)	0.728
PTCA, n (%)	17 (39)	11 (33)	6 (54)	0.211
NA (μg/kg/min, first 72 h), median (IQR)	0.25 (0.44)	0.37 (0.54)	0.12 (0.13)	0.006
ADRE, (μg/kg/min, first 72 h), median (IQR)	0.02 (0.13)	0.06 (0.20)	0.00 (0.04)	0.043
Total catecholamines (NA + ADRE, first 72 h)	0.36 (0.73)	0.54 (0.67)	0.12 (0.16)	0.002
Cristalloid (ml/kg/h, first 72 h), mean (SD)	6.0 (9.8)	7.3 (11.2)	2.4 (0.8)	0.023
Packed RBC (n, first 72 h), mean (SD)	5.2 (4.7)	5.5 (4.8)	4.4 (4.4)	0.449
FFP (n, first 72 h), mean (SD)	2.4 (3.0)	2.6 (3.1)	1.6 (2.7)	0.294

*ADRE* adrenaline, *BP* blood pressure, *ECMO* extra-corporeal membrane oxygenation, *FFP* fresh frozen plasma, *ICU* intensive care unit, *IHCA* in-hospital cardiac arrest, *LOS* length of stay, *NA* noradrenaline, *OHCA* out-of-hospital cardiac arrest, *PEA* pulseless electrical activity, *PTCA* percutaneous transluminal coronary angioplasty, *RBC* red blood cells, *SAPS* simplified acute physiology score, VF ventricular fibrillation

**Fig. 1:**
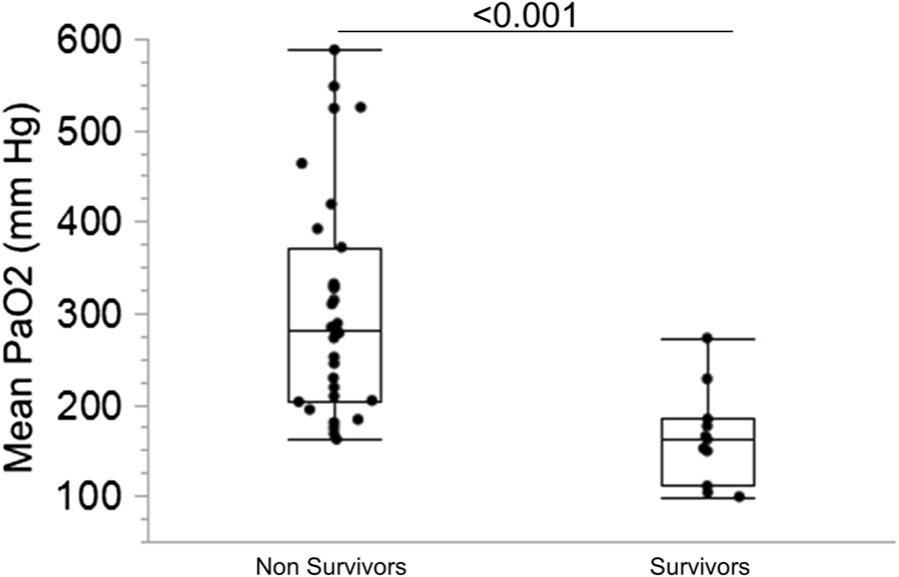
Comparison of mean 24 h PaO_2_ in survivors and non survivors

**Fig. 2:**
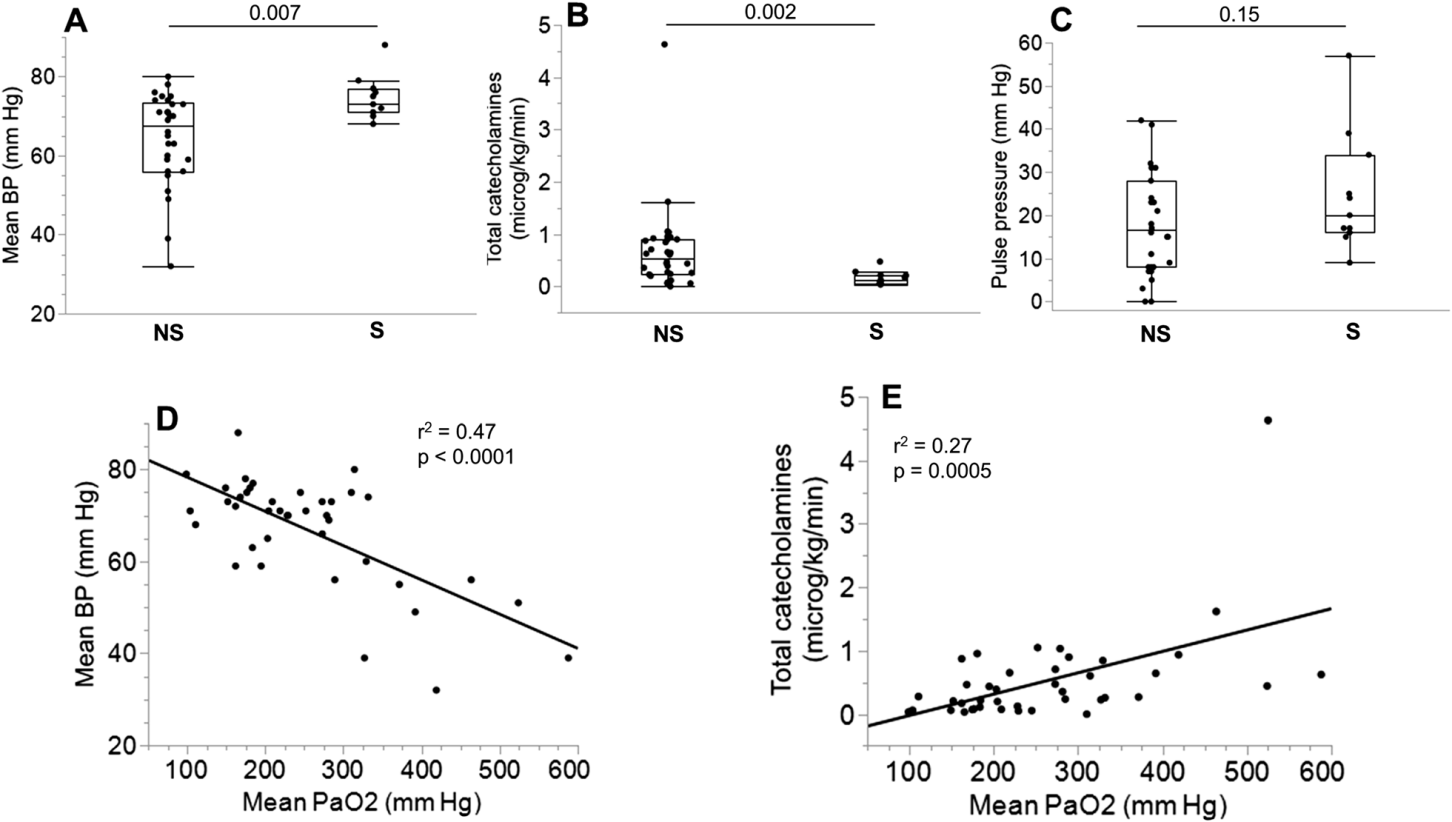
Comparison of hemodynamic variables and vasopressor therapy in survivors and non survivors during ECPR and correlations with mean PaO_2_. **A** Mean blood pressure during the first 24 h of ECPR; **B** Total catecholamines administered during the first 72 h of ECPR; **C** Pulse pressure (systolic minus diastolic blood pressure) during the first 24 h of ECPR; **D**, **E** simple linear regressions showing correlations between mean PaO_2_ and mean blood pressure (**D**) and catecholamine therapy (**E**) during ECPR. Box plots show median, 1st and 3rd quartiles, whiskers indicate minimal and maximal values. *NS* non survivors, *S* survivors

In two patients dying early after ECPR initiation, arterial blood samples were not obtained. Non-survivors had significantly higher mean PaO_2_ during the first 24 h (Table 1 and Fig. 1). At each time point, the values of PaO_2_ in non-survivors vs survivors were: ECMO initiation: 444 ± 107 vs 352 ± 136 mmHg (p = 0.03); 2 h: 287 ± 175 vs 164 ± 108 mmHg (p = 0.07); 6 h: 253 ± 148 vs 125 ± 78 mmHg (p = 0.02); 12 h: 161 ± 111 vs 85 ± 22 mmHg (p = 0.004); 24 h: 121 ± 44 vs 94 ± 21 mmHg (p = 0.02). At the respective time-points, the number of measurements were 11 in survivors and 31, 27, 27, 22 and 18 in non-survivors. Non-survivors also displayed lower mean blood pressure during the first 24 h, and received significantly more vasopressive catecholamines (Table 1 and Fig. 2A, B). Pulse pressure during the first 24 h was not different between non-survivors and survivors (Table 1 and Fig. 2C). The mean PaO_2_ value during the first 24 h displayed a significant negative correlation with mean blood pressure (Fig. 2D) and positive correlation with total amount of vasopressors (NA + Adre, in μg/kg/min during the first 3 days, Fig. 2E).

Variables associated with in-hospital mortality at the 5% level in univariate analysis (Table 2) included the duration of low flow, mean PaO_2_, the first arterial lactate concentration, the amounts of catecholamines and IV fluids administered, as well as mean blood pressure during the first 24 h. Table 3 shows the results of the two models of multivariable logistic regression analyses, which indicate that mean PaO_2_ was the only co-variate showing a p value < 0.05 for the association with mortality in both models. The results of the logistic regressions including only 2 co-variates (mean PaO_2_ and low flow; mean PaO_2_ and no flow; mean PaO_2_ and initial rhythm), also show an association between mean PaO_2_ and mortality following Bonferroni correction.

**Table 2:** Variables associated with in-hospital mortality (univariate analysis)

Variable	OR [95% CI]	p value
Age	1.03 [0.98–1.09]	0.244
Localization of CA (OHCA vs IHCA)	0.88 [0.20–3.54]	0.858
Initial rhythm (VF vs non VF)	0.40 [0.08–1.73]	0.226
SAPS II	1.06 [0.99–1–15]	0.052
No flow duration	0.91 [0.66–1.26]	0.580
Low flow duration	1.06 [1.02–1.11]	0.003
Lactate at ECMO initiation	1.22 [1.06–1.46]	0.005
Mean PaO_2_ first 24 h on ECMO	1.03 [1.01–1.06]	< 0.001
Mean PaCO_2_ first 24 h on ECMO	0.95 [0.82–1.08]	0.462
Mean BP (first 24 h)	0.84 [0.72–0.98]	0.001
Pulse Pressure (first 24 h)	0.96 [0.90–1.01]	0.129
Total catecholamines (NA + ADRE)	4.51 [1.70–15.68]	0.012
Total Cristalloid	1.67 [0.96–2.89]	0.005
Catheter position (right RA vs other)	2.10 [0.53–9.54]	0.297
Coronarography	0.78 [0.32–5.23]	0.728
PTCA	0.42 [0.10–1.67]	0.217
Packed RBC	1.06 [0.91–1.26]	0.459
FFP	1.14 [0.87–1.49]	0.322

Univariate analysis of variables associated with in-hospital mortality. Data are shown as p values and odds ratio (OR) with 95% confidence interval (CI)

*ADRE* adrenaline, *CA* cardiac arrest, *ECMO* extra-corporeal membrane oxygenation, *FFP* fresh frozen plasma, *IHCA* in-hospital cardiac arrest, *NA* noradrenaline, *OHCA* out-of-hospital cardiac arrest, *PTCA* percutaneous transluminal coronary angioplasty, *RA* radial artery, *RBC* red blood cells, *SAPS* simplified acute physiology score, *VF* ventricular fibrillation

**Table 3:** Variables associated with in-hospital mortality (multivariable analysis)

Variable	Odds ratio	95% CI	p value
*(a) Model using co-variables significantly associated with mortality in univariate analysis*			
Low flow duration	1.03	0.96–1.11	0.39
First Lactate	1.19	0.92–1.53	0.18
Mean BP	0.86	0.69–1.07	0.17
Mean PaO_2_	1.02	1.01–1.05	0.02
*(b) Model using co-variables frequently associated with mortality in univariate analysis*			
Age	1.04	0.92–1.17	0.55
No Flow duration	0.01	0.01–1.38	0.09
Low flow duration	1.26	0.99–1.59	0.06
ECMO duration	1.01	0.99–1.03	0.25
Shockable rhythm	0.01	0.01–3.29	0.12
Localization (IH vs OH)	0.05	0.01–7.32	0.24
Mean PaO_2_	1.07	1.01–1.13	0.03
*(c) Models with 2 co-variables*			
Mean PaO_2_	1.04	1.02–1.08	0.009
﻿No flow	0.62	0.31–1.07	0.121
Mean PaO_2_	1.02	1.01–1.06	0.011
Low flow	1.03	0.98–1.11	0.232
Mean PaO_2_	1.03	1.01–1.06	0.013
Shockable rhythm	0.42	0.06–2.86	0.383

*CI* confidence interval. Odds ratio for continuous variables are calculated per unit change for each variable (1 mmol/L for lactate, 1 mmHg for mean BP, 1 y for age, 1 min for no flow and low flow duration, 1 h for ECMO duration, 1 mmHg for mean PaO_2_)

**Fig. 3:**
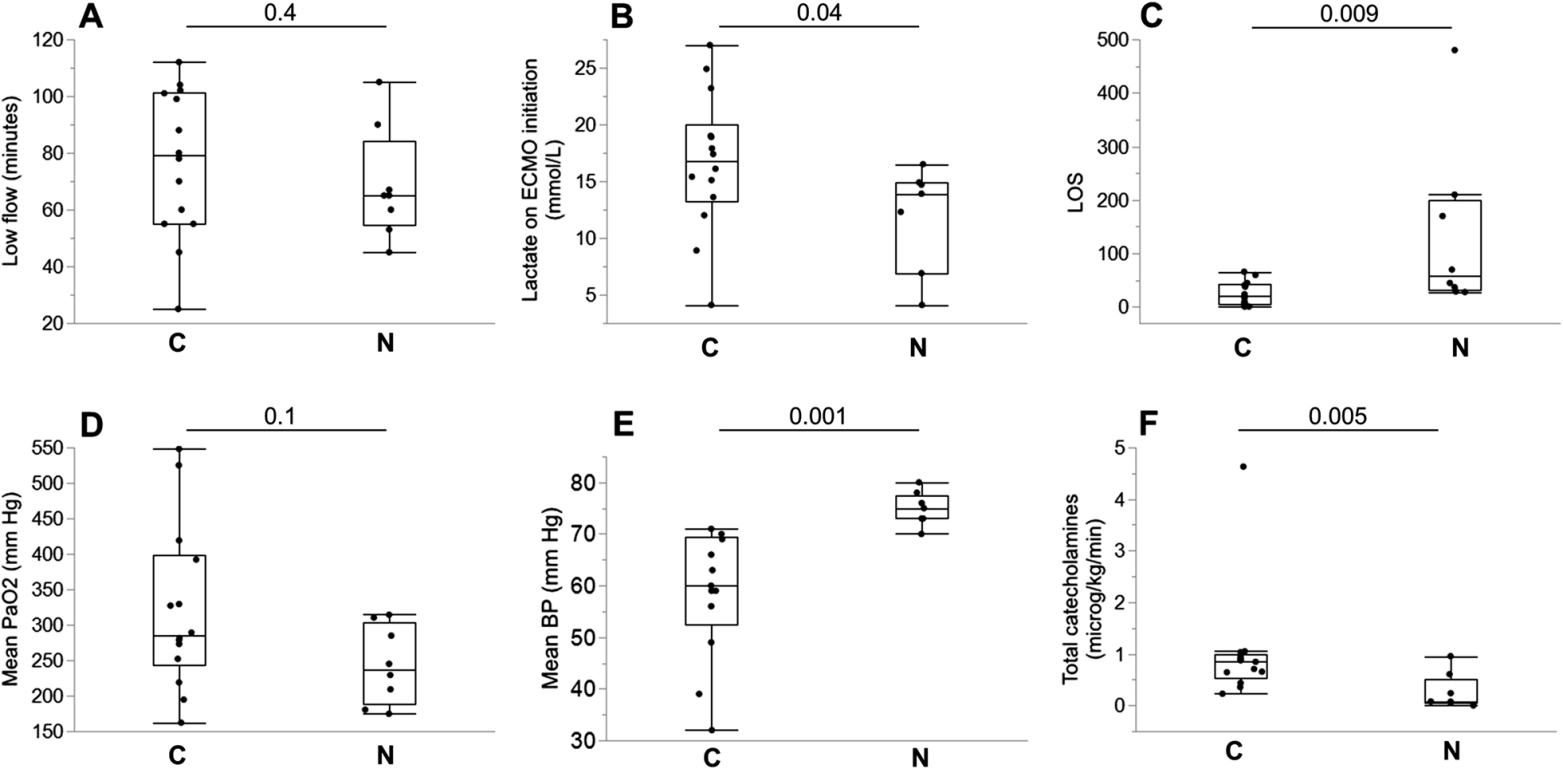
Characteristics of patients dying from circulatory or neurological cause during ECPR. **A** Duration of low flow; **B** first value of arterial lactate after ECPR initiation; **C** length of stay (LOS) in the ICU; **D** mean PaO_2_ during the first 24 h of ECPR; **E** mean blood pressure during the first 24 h of ECPR; **F** total catecholamines administered during the first 72 h of ECPR. On the X-axis, C means death from circulatory failure and N means death from neurological causes. Box plots show median, 1st and 3rd quartiles, whiskers indicate minimal and maximal values

The causes of refractory CA included acute myocardial ischemia (n = 24, 55% of patients), acute hypoxic CA (n = 4, 9%), primary arrhythmia (n = 3, 7%), acute pulmonary embolism (n = 3, 7%), toxic/metabolic origin (n = 3, 7%, including one cocaine intoxication, one aluminium phosphorus intoxication and one severe metabolic acidosis), heart transplant rejection (n = 2; 4%), undetermined cause (n = 2, 4%), and other causes (n = 3, 7%, including one intraoperative CA, one pericardial tamponade and one subarachnoid hemorrhage). The two main causes of death under ECPR were profound circulatory shock (n = 15, 46% of patients) and neurological injury (severe anoxic encephalopathy or major stroke, n = 8, 24% of patients). Other causes of death included hemorrhagic shock (n = 3). Multiple organ failure (n = 4), septic shock (n = 2) and technical issue (n = 1, related to an intractable failure to obtain an adequate venous inflow, possibly due to extensive thrombosis in the inferior vena cava). Comparisons between patients dying from circulatory or neurological causes are shown in Fig. 3. Low flow was not statistically different in the two groups (Fig. 3A). Patients dying from circulatory cause had significantly higher initial lactate (Fig. 3B) and shorter ICU LOS (Fig. 3C). The mean PaO_2_ during the first 24 h was not different (p = 0.1 between both groups, Fig. 3D). Patients with neurological death had significantly higher mean BP during the first 24 h (Fig. 3E) and received significantly less vasopressive catecholamines (Fig. 3F).

**Fig. 4:**
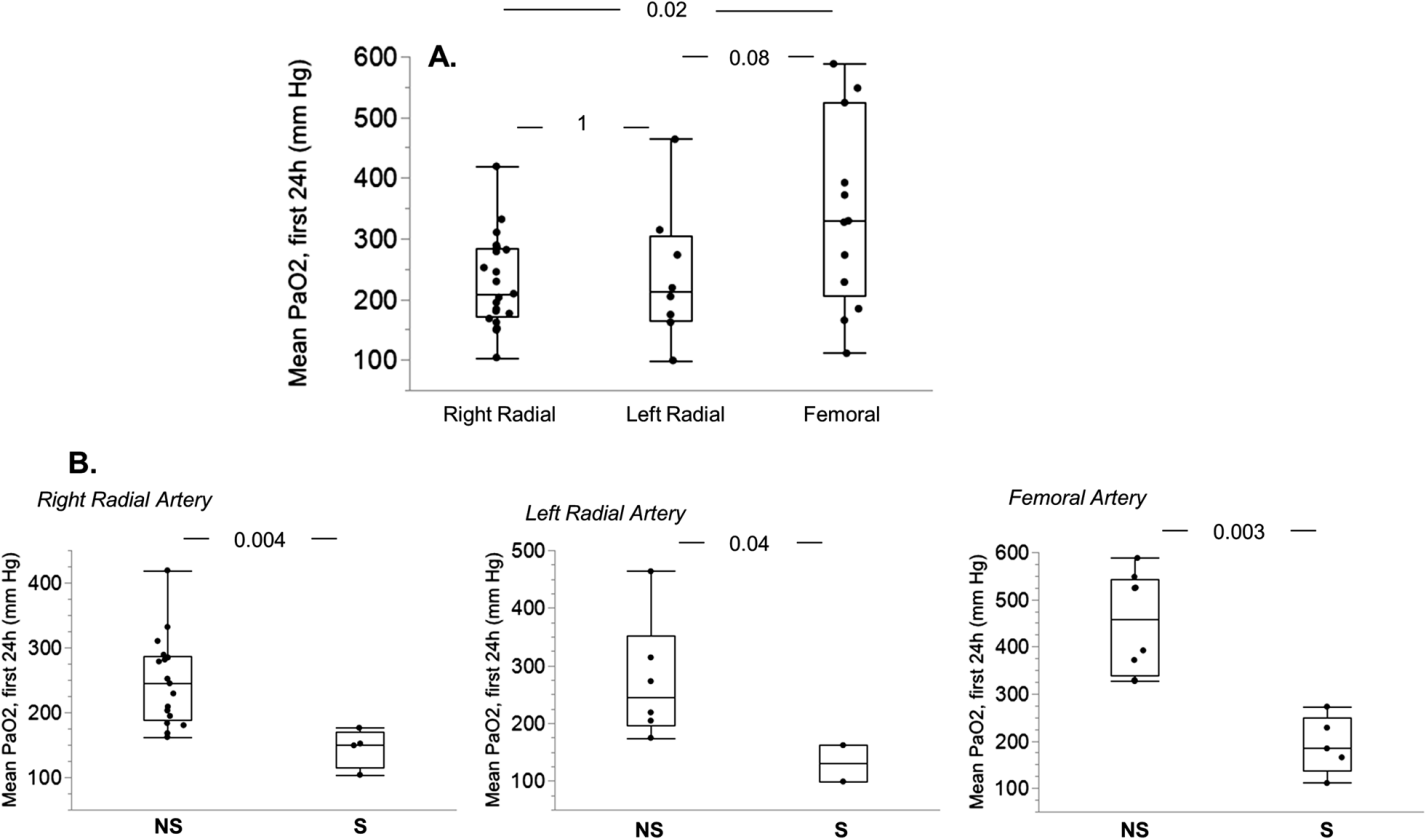
Mean PaO_2_ values according to the site of arterial blood sampling. **A** Mean PaO_2_ during the first 24 h of ECPR according to the position of the arterial catheter in the whole cohort; **B** mean PaO_2_ values in survivors and non survivors according to the site of blood sampling (right radial, left radial or femoral artery). Box plots show median, 1st and 3rd quartiles, whiskers indicate minimal and maximal values. *NS* non survivors, *S* survivors

Mean PaO_2_ measured via a right arterial catheter was not different from that measured from a left radial catheter, but was lower than that measured via a femoral catheter, and mean PaO_2_ measured from a left radial catheter was not different from that measured via a femoral catheter (Fig. 4A). When comparing mean PaO_2_ between survivors and non survivors according to the catheter position, values were significantly lower in survivors for each position of the arterial catheter (Fig. 4B).

## Discussion

### Background and previous work

The restoration of systemic oxygenated blood flow after a prolonged period of hypoxia may trigger widespread reperfusion injury. A key mechanism of reperfusion injury is the generation of oxidants and free radicals [19], whose flux increases in proportion with the local PO_2_ [20–22], implying an increased risk of oxidant-mediated damage at higher PO_2_ during reperfusion [23]. In the setting of ECPR, the risk of hyperoxia is particularly elevated, due to the ease of oxygenating blood through the membrane oxygenator [5, 12], and a few retrospective studies have indeed reported a negative impact of hyperoxia on survival after ECPR [13, 14, 24]. In a retrospective cohort of 291 ECPR patients, Chang et al. reported that a first PaO_2_ value during the first 24 h after ECPR initiation between 77 and 220 mmHg, was significantly associated with neurologically intact survival in comparison to higher values [14]. In a retrospective cohort of 66 patients undergoing ECPR for refractory OHCA, Halter et al. reported a significant association between PaO_2_ measured at 30 min after ECPR initiation and 28 days mortality. Using a threshold value of PaO_2_ of 300 mmHg to define hyperoxia, the odds ratio for 28 days mortality was 4.07 [13]. In our study, non survivors displayed a higher mean 24 h PaO_2_, lower mean blood pressure, higher needs in vasopressors, and profound circulatory shock was the primary cause of death in a majority of non survivors. Taken together, these findings suggest that severe vascular failure with refractory circulatory shock may represent an important mechanism of hyperoxia toxicity during ECPR.

### Hyperoxia toxicity in ECPR

Hyperoxia results in an increased vascular generation of superoxide (O_2_^.−^), which reacts rapidly with nitric oxide (NO^.^) to form peroxynitrite, that can trigger significant vascular contractile failure through a number of processes [25–27]. In addition, oxidants such as peroxynitrite promote the expression of multiple inflammatory cytokines and mediators [28–31], which also reduce vascular tone and may precipitate hypotension. These effects may contribute to foster a sepsis-like state, characterized by an irreversible loss of vascular contractility and refractory hypotension with negative prognostic impact after prolonged resuscitation and ECPR, as recently reported by Jouffroy et al. [32]. Obviously, the formation of peroxynitrite and other oxidants, together with the generation of inflammatory mediators, at different levels of PaO_2_ during ECPR, should be evaluated in future investigations to explore these mechanisms.

### Importance of the localization of arterial blood sampling in ECPR

A critical aspect in the interpretation of arterial blood gases during peripheral VA-ECMO is the localization of arterial blood sampling and right radial artery sampling is recommended [33], owing to the risk of upper body hypoxia in case of cardiac recovery and impaired pulmonary gas exchange [34]. Accordingly, we found that PaO_2_ was lower when measured from a right radial artery than from a femoral artery, while it did not differ from values obtained from the left radial artery. One could therefore argue that the lower PaO_2_ in survivors might reflect an earlier recovery of native cardiac function, but this appears unlikely in view of the similar values of pulse pressure, an indirect, real-time measure of native cardiac output during extracorporeal support [35], in survivors and non survivors.

### Main causes of deaths during ECPR

The two main causes of death were early circulatory failure and delayed neurological damage. Patients dying from either cause had similar durations of low flow and mean PaO_2_, but initial lactate levels were lower in patients dying from neurological damage, pointing to less profound systemic anoxia. These patients also had higher mean blood pressure and required significantly less vasopressors. Overall, these findings suggest that patients with more severe anoxia (higher lactate levels) might develop more severe reperfusion injury under hyperoxic conditions, leading to predominantly vascular failure and early deaths, whereas those with less severe anoxia would survive the early stage and develop delayed hyperoxic neurological damage. This hypothesis should require validation in larger cohorts of patients treated with ECPR for refractory cardiac arrest.

### PaO_2_ is the main variable associated with mortality in ECPR

We did not find significant association between the initial rhythm and survival, which differs from the notion that survival under ECPR is better in patients with an initial shockable rhythm [36]. This discrepancy most likely reflects the small sample size in our study. Only 52% of patients had an initial shockable rhythm, and among those with a non-shockable rhythm, all patients with asystole died, whereas 3 out of 14 patients (21%) with pulseless electrical activity (PEA) survived to hospital discharge. Interestingly, these 3 patients had the lowest mean PaO_2_ (152, 162 and 184 mmHg, respectively) among the 14 PEA patient, which may suggest that the avoidance of hyperoxia during ECPR could be critical to determine outcome in PEA patients undergoing ECPR.

Besides PaO_2_, survival in our cohort was significantly associated with the duration of low flow in univariate analysis, in line with previous investigations [37–39]. However, this association was not observed in a multivariable analysis evaluating several co-variates commonly associated with poor outcome after CA and ECPR, such as no flow, low flow, initial rhythm, localization of CA (IHCA vs OHCA) and age, together with mean 24 h PaO_2_. We also included the duration of ECMO as a co-variate, given that the initial sweep gas oxygen fraction was set at 100%, which could have favored persisting hyperoxia in these patients. With the exception of mean PaO_2_, none of these variables displayed a significant association with mortality. Although these findings may suggest a particularly negative prognostic implication of hyperoxia in ECPR, they warrant cautious interpretation. Owing to the relatively small sample size in our study, the results of multivariate analysis may be subject to some bias related to an overestimation of regression coefficients [18]. For this reason, we performed additional analyses using only one co-variate with mean PaO_2_, including no flow, low flow and initial rhythm, which confirmed the association of mean PaO_2_ with mortality. In addition, due to the retrospective nature of our study, the association of PaO_2_ with mortality could represent a surrogate for the sickness of patients. Physicians would indeed be inclined to maintain high levels of administered O_2_ to the most severely affected patients, and hyperoxia would therefore be a side effect of such management.

### Study limitations

Our study has several limitations. First, we must acknowledge the usual limitations related to the retrospective design of our study [40] and to its relatively small sample size. Second, although we established an association between hyperoxia and mortality, as well with refractory circulatory failure, such associations do not necessarily mean a causal relationship. Third, the absence of internal recommendations for the management of arterial oxygen levels during ECPR may have favored the development of hyperoxia, which could have been avoided with a dedicated clinical protocol. Fourth, we relied on pulse pressure as an indirect evaluation of native cardiac output, which may be of limited reliability under conditions of varying cardiac loading conditions, especially afterload [41]. Direct assessment of native cardiac function using echocardiography would have been more appropriate in this setting, but we do not routinely perform echocardiography in the first 24 h after ECPR initiation. Fifth, we did not measure circulating mediators related to oxidative stress and inflammation, which could have given important insights on the effects of hyperoxic reperfusion during ECPR. Such measurements will be the matter of additional future investigations.

## Conclusion

In conclusion, although we acknowledge several important limitations, our study shows that, in patients undergoing ECPR for refractory cardiac arrest, a high PaO_2_ during the first 24 h of support is associated with worse outcome, possibly by promoting severe vascular failure and refractory circulatory shock. PaO_2_ should therefore be strictly controlled during the first 24 h of ECPR, via blood sampling from the right radial artery. Future studies should evaluate the impact of different PaO_2_ levels during ECPR on biomarkers of oxidative stress and inflammation, to provide further insights into possible mechanisms of hemodynamic collapse in this setting.

## Supplementary Information

**Additional file 1:Supplementary Table 1.** Study data set.

## Data Availability

All data generated or analysed during this study are included in this published article and its Additional file 1: Table 1.

## Abbreviations

ECMO: Extra-corporeal membrane oxygenation
VA-ECMO: Veno-arterial extracorporeal membrane oxygenation
ECPR: Extracorporeal cardiopulmonary resuscitation
CA: Cardiac arrest
OHCA: Out-of-hospital cardiac arrest
IHCA: In-hospital cardiac arrest
ROSC: Return of spontaneous circulation
ECLS: Extracorporeal life support
NA: Noradrenaline
Adre: Adrenaline
IV: Intravenous
PEEP: Positive end-expiratory pressure
BP: Blood pressure
PTCA: Percutaneous transluminal coronary angioplasty
FFP: Fresh frozen plasma
RBC: Red blood cells
RA: Radial artery
SAPS: Simplified acute physiology score
VF: Ventricular fibrillation
LOS: Length of stay
